# Contested discourses and culture sensitivity: Norwegian nursing students' experience of clinical placement in Nicaragua

**DOI:** 10.1002/nop2.114

**Published:** 2017-12-09

**Authors:** Solveig Kirsti Grudt, Hans Hadders

**Affiliations:** ^1^ Faculty of Medicine and Health Sciences Department of Public Health and Nursing Norwegian University of Science and Technology Trondheim Norway

**Keywords:** clinical placements, cultural competence, culture sensitivity, discourses, nurse education

## Abstract

**Aim:**

The purpose of this study was to gain understanding of Norwegian students' practical experience of “culture sensitivity.”

**Design:**

Using focus‐group interviews and individual written assignments, we draw on a Foucauldian‐inspired approach to analyse nursing students' narratives about their clinical placement in Nicaragua.

**Method:**

Seven third‐year bachelor nursing students enrolled in a clinical placement programme on the Caribbean coast in Nicaragua and participated in focus‐group interviews. Interviews were conducted prior to their departure to Nicaragua and after their return to Norway. Other sources of data included learning objectives for clinical placement, written individual assignments with students' reflections about their experiences and achievement of learning objectives.

**Results:**

Students expressed gradually increased awareness about the nursing discourses and power relations shaping clinical encounters throughout their learning trajectory in clinical placement. They became more aware of the politics of nursing practices through their experiences of clashes between different nursing discourses.

## 1 INTRODUCTION

In the wake of globalization and a focus on global health, international clinical placement experience in low‐ and middle‐income countries has become a popular strategy to enhance culture sensitivity and cultural competence among nursing students. “Cultural awareness” and “cultural competence” are widely recognized as an essential component for nursing students in contemporary multicultural societies to tackle various challenges due to cultural difference encountered in the clinic (Alpers & Hanssen, 2014; Jørgensen & Hadders, 2015; Reid‐Searl et al. 2010; Maltby & Abrams, 2009; Tabi & Mukherjee, 2003).

Followers of a transcultural nursing approach advocate culture competence and invoke culture sensitivity in nursing. However, in a review of transcultural nursing relevant to the British context, Narayanasamy and White (2005) point out that Leininger's transcultural nursing model is inadequate, dated, based on the idea of free will and an assumption that all good nurses want to provide “culturally specific” care. Narayanasamy and White argue that institutional racism pervades health care, including nursing (Narayanasamy & White, 2005, p. 106). Culley (2006) underscores that among advocates of the transcultural nursing approach, there is often a lack of awareness of the political and social context embedded in “cultural” aspects in the clinic and a failure to theorize power relations. Culley advocates a revised version of the culture concept with a focus on social processes and the fluidity of ethnic identities (Culley, 2006). Any further elaboration and discussion about the revised culture concept fall outside the scope of this paper. In a recent publication, Thomas Foth presents an extension of this criticism and argues that the concept of care in most “newer” nursing theories obscures the political agenda of nursing and fails to provide a critical framework to analyse nursing practice (Foth, 2013). Using a Foucauldian theoretical framework, Foth claim, “These theoretical approaches neglect the fact that nursing is above all a profession with a societal task and is characterized by an asymmetrical power relation between nurses and their patients” (Foth, 2013, p. 284).

Nevertheless, there is an agreement that international placements can provide an important awakening, which can lead to further awareness about sociocultural differences. Several authors underscore that education about ethnicity, racism, sociopolitical perspectives and social determinants of health are crucial for students to make sense of their experiences (Alpers & Hanssen, 2014; Culley, 2006; Harrowing, Gregory, O'Sullivan, Lee, & Doolittle, 2012; Mkandawire‐Valhmu & Doering, 2012; Racine & Perron, 2012). Following up on the critique presented above, we argue that an awareness of the political and social context can be fostered and enhanced through various supportive educational interventions during the entire graduate educational process. In this paper, we present the results of our study and we illustrate our position empirically as well as theoretically. First, a brief background to nurse education in Norway is given. Second, the theoretical approach to power, knowledge and discourses is explained. Third, the design of the study is outlined followed by findings, interpretation and a discussion. The article ends with some concluding remarks.

### 1.1 Nurse education in Norway

Clinical placement is an important arena for undergraduate nursing education. Access to the clinic gives students an opportunity to acquire practical skills and contextualized knowledge in bedside situations. In the European context, nursing training and clinical placement traditionally took the form of an apprenticeship model where students spent most of their time in a supernumerary capacity working alongside qualified nurses in hospital wards. Such knowledge transfers were typically hierarchical, with little personal supervision and often based on unreflective copying of the role model's task performance (Scott, 2013; Spouse, 1998, 2001). Clinical placement in current Nicaraguan nursing education is akin to the traditional apprenticeship model.

Clinical practicum has been part of nursing education since the establishment of institutional nursing training in Norway. At present, approximately 50% of the nursing training constitutes clinical placement (National Curriculum for Nursing Education 2008). In Norway, at present, Norwegian nursing students are currently allotted a personal clinical mentor and a ward nurse, who are responsible for supervision and guidance during clinical placement. Mentors are important guides and gatekeepers to their professional communities (Scott, 2013; Spouse, 2003).

The current Norwegian nursing educational paradigm has a strong focus on academic, analytical, assertive and reflective qualities (National Curriculum for Nursing Education 2008). A common strategy to support reflective qualities and bridge the potential gap between theory and practice in nursing education has been the application of problem‐based learning and active reflection during clinical placement (PBL). Student active learning methods, reflexion, self‐directed learning and PBL are regular features of the bachelor nursing programme at the Norwegian University of Science and Technology, Faculty of Medicine and Health Sciences, Department of Public Health and Nursing, Faculty. One of the objectives in the current National Curriculum for Nursing Education is respect for fundamental human rights. The Norwegian nursing education has a strong emphasis on the integrity of the patient and equality as a fundamental principle of health care (National Curriculum for Nursing Education 2008). Students learn to respect equality as a founding principle during their primary education and as they are socialized into the Norwegian welfare system and ideology. Nursing theories taught to Norwegian bachelor nursing students often tend to present empathetic care as an essential part of nursing practice.

### 1.2 Norwegian Nursing students' clinical placement in Nicaragua

In January 2016, seven third‐year bachelor‐nursing students from NN, Department of Public Health and Nursing spent 10 weeks in different health institutions and a local hospital at the Caribbean coast. Prior to departure, the seven students participated in an obligatory bachelor course in Global Health, consisting of five international credit points (ECTS) and they also received an elective course organized by supervisors at the nursing college. The latter course was meant to give the group some knowledge and tools when preparing for their placement. Among other things, students were briefed through lectures and publications about disease panorama, Nicaraguan sociopolitical system, various local health challenges, the nursing role in Nicaragua and personal health and safety. The elective course for International clinical placement had a scope of 15 credit points (ECTS). This course had the following major learning objectives; cultural sensitive nursing and nursing role in a global perspective. The learning objectives aimed at the following skills; nursing practice in a multicultural and multidisciplinary perspective, communication in a multicultural and multidisciplinary perspective, culture sensitivity in nursing practice, being innovative and solution oriented in a new cultural environment. The general competence focused on reflection about professional practices, ethical issues and adaptability to new situations in the clinical placement (HSYK304P: Optional Course for clinical placement in Global Health (2016). The Nicaraguan organization facilitated students with group supervision and regular debriefing outside the ward by a clinical educational nurse consultant once a week during clinical placement. Weekly Skype meetings and frequent email communication with their Norwegian teachers were also held.

### 1.3 The Nicaraguan context

The seven nursing students completed their clinical placement in one of two of Nicaragua's autonomous regions, located in the south at the Caribbean coast. This region is called La Región Autónoma del Atlántico del Sur (RAAS) and this area together with La Región Autónoma del Atlántico del Norte (RAAN) has the highest percentage of rural population in the country. RAAN the region in the north also has the four poorest municipalities (Krasnoff, 2013). The Nicaraguan Ministry of Health (MINSA) launched the following slogan in 2008; “Health, a right of all, an investment in development” (Krasnoff, 2013). In the RAAN region, health for all means to provide health care for all the different ethnic groups including afro‐descendants (creoles), mestizos and various other indigenous groups. In 2007, approximately 25% of the 2400 medical physicians worked in Managua, the capital of Nicaragua. There was approximately one nurse per 909 inhabitants in Nicaragua the same year. In RAAS, where the students did their clinical placement, nurses and nurse auxiliaries, mainly women, represent 74% of the health work force. System of the Locally Integrated Health Services (SILAIS) manage the public hospitals and community clinics in the 17 regions in Nicaragua and is administrated by MINSA. Health challenges for the two regions where Department of Public Health and nursing, Faculty of Medicine and Health Sciences, Norwegian University of Science and Technology send the bachelor nursing students are a higher maternal mortality rate than the rest of the country, a higher incidence of tuberculosis and malaria, mostly among the indigenous population. Access to mental health services is limited with only one psychiatrist for the entire RAAS and RAAN regions (Krasnoff, 2013).

### 1.4 Theoretical approach to power, knowledge and discourses

In this study, we draw on a Foucauldian approach to uncover nursing students' narratives about their clinical placement in Nicaragua. We use the concepts “discourse” and “power,” coined by Foucault (1972), to explore the contesting discourses about nursing practice encountered by the students. With the help of these concepts, we highlight the political and social aspects of nursing in the clinic. With this approach, we aspire to explore power relations in nursing practice and illuminate “culture sensitivity” as it emerges in students' narratives during their learning trajectory.

### 1.5 Discourse

“A discourse is a systematic body of text, speech and action regarding a specific subject are in the realm of human experience that mobilizes power in the form of productive knowledge in the social world” (Powers, 2013, p. 6). To uncover the power of discourses in any setting, one has to investigate the politics of everyday social practice. Springer and Clinton (2015) underscore that Foucauldian analysis is a methodology well suited for an inquiry of nursing knowledge, power, practice and organization.

### 1.6 Power and knowledge

The major part of French philosopher‐historian Michel Foucault's legacy has been a persistent critique of the modernist enterprise through what he initially called an “archology of knowledge” with the goal of uncovering the historic relationship between power and knowledge (Foucault, 1972). Foucault underscored that to understand how discourses evolve and are produced, it is crucial to understand the “power‐knowledge” dyad that created these discourses and sustain them. As an historian, Foucault explored the genealogical legacy and historical contingency of discourses and discursive practices over time. Furthermore, Foucault linked the production of power to particular constructions of established forms of knowledge and institutionalized practices (Foucault, 1972, 1973). For instance, Foucault mental illness is not an entity discovered by “objective” psychiatric science; on the contrary, mental illness is a product of the social practice of psychiatric science. In other words, the discourses of psychiatry create and controls psychiatric conditions by defining and treating these (Powers, 2013, p. 6). Thus, Foucault claimed that a conception of professional knowledge or claim to defensible truth or knowledge is closely tied to the social practice and power of the professional institutions where these conceptions are conceived (Springer & Clinton 2015, pp. 89–90). In modern medicine, biomedical ordering and surveillance are common and mostly taken for granted. The process of “objectification,” whereby the patients are abstracted from the disease through the “clinical gaze” by professionals reading signs of the physical body, is analysed by Foucault in his work, The Birth of the Clinic (Foucault, 1973).

Unlike common understanding of power, typically tied to juridical enforced sanctions trough penal systems and proceeded by brute physical force from top to down, Foucault's understanding of power differ, in several respects. Power operates everywhere in subtle ways in tiny complex ramifications at the level of local practices of everyday life. Penny Powers underscore that power is not intentional, can have unintended consequences and “must be understood as a network on interacting forces that are goal‐driven, relational and self‐organized. Power creates tensions between, within and among individuals in a group” (Powers, 2007, p. 28).

The notion of power, the power to know and the power to control is the most important notion in Foucault's work because it constitutes the basis for the understanding and analysis of discourses (Powers, 2007; p. 28). Penny Powers underscore that wherever there is power, there is resistance. These two forces are always found together and are constituted by each other (Powers, 2007, p. 31). Various discourses avail different subject positions. It is not possible to act outside a given discourse.

In this paper, we focus mainly on the third part of discourse analysis, based on Rawlinson as described by Powers, that is, the “power analysis” of current power relations between people in the clinical setting. Due to the limited scope of this paper, we do not analyse the historical genealogy and wider structural aspects of discourses (Powers, 2013, p. 11). With the help of a Foucauldian approach, we highlight some social and political aspects of nursing practice, in Norway and Nicaragua.

## 2 THE STUDY

### 2.1 Aim

The purpose of this study was to gain understanding of students' practical experience of “culture sensitivity.”

### 2.2 Data collections and methods

This study was exploratory, using qualitative data. Seven third‐year bachelor students enrolled in a clinical placement programme in Nicaragua were invited to participate in focus‐group interviews (Barbour, 2010; Halkier, 2010). Data were collected from 19 November 2015 to 14 April 2016. Focus‐group interviews were conducted prior to their departure to Nicaragua and after their return to Norway. All students agreed to participate. Interviews were approximately 90 min in length. The first author moderated the focus groups and the second author assisted. The interviews were facilitated by open‐ended questions with low moderation, and all participants had an opportunity to vent their anticipations, views and practical experiences of culture sensitivity. Participants were asked to describe their expectations, experiences of sociocultural encounters and how they learnt and coped with challenges and the impact of their clinical placement. More specifically, in the first interview, students were asked about their experience with cross‐cultural encounters, their expectations about Nicaraguan nursing practice and their understanding of culture sensitivity in professional nursing practice. In the second interview, after students′ return to Norway, they were asked about the challenges they had experienced during clinical placement and their reflections about achieving their learning objectives (Halkier, 2010).

Other sources of data included learning objectives for clinical placement, written individual assignments with reflections about students' experiences and achievement of learning objectives. Students submitted the first assignment during the first weeks of clinical placement. This assignment consists of a short approximately 700 word narrative describing their feelings, thoughts and reactions towards a challenging situation they experienced during their clinical placement. The students were asked to describe their considerations and dilemmas. The aim of this assignment was to reflect on their own and others' nursing practice in a sociocultural context. The second written assignment was submitted at the end of their clinical placement. This written assignment consists of a longer approximately 2500 word paper. In this assignment, students were asked to reflect on their learning objectives from their preparatory courses and to what extent they had achieved these. They were also asked to reflect on expectations and goals related to their clinical practice.

### 2.3 Ethical considerations

Approval for the study “Learning in clinical placement abroad. Culture sensitivity, cultural competence and safe nursing practice” was obtained from the Norwegian Centre for Research Data (NSD). An ethical issue was the potentially biased relationship between the researcher/teacher and students. This issue was managed by presenting written and oral information about the study to students before recruitment. Participation was voluntary and withdrawal from the study at any time had no consequence for the students. Written informed consent was sought for interviews, learning objectives and written individual assignments. In addition, all students were provided with written information about the study and the conditions for their involvement. Participants were assured that no individual names would be used in any reports of the study. Code number identified the transcripts. Student assignments and written individual learning objectives were rendered anonymous.

### 2.4 Data analysis and methodological considerations

The moderator transcribed the interviews. Transcriptions as well as audio version of interviews have been used during analysis of the interviews (Strauss & Corbin, 1990). The authors have read learning objectives, students′ written assignments and transcripts of the interviews several times independently. The authors coded the various data sources by hand and content analysis was used to identify major themes and patterns (Kvale, 2009). We have taken considerable time to decide on an analytical approach. Initially, the incentive to use discourse as an analytical concept came from the students' forceful narratives about contested nursing practices. We have done extensive reading and research to find a suitable way to apply Foucault without overdetermining our data and immobilizing the valuable narratives of our informants. We also have considered the danger of alienating our targeted readers (e.g. nurses and educators in the field) with an overly academic approach. That is why we have limited the use of Foucauldian terminology to a minimal extent.

## 3 FINDINGS

We analysed the data material with the Foucauldian‐inspired approach presented above. Themes were identified in several different phases of the clinical placement and learning process, prior to departure, during clinical placement and after returning to Norway. We have highlighted the various data sources (e.g. first focus‐group interview, first assignment, second assignment and second focus‐group interview) used and presented throughout the findings section. Thus, findings represent the students' learning trajectories in real time. Major themes were apprehension about conflicting nursing discourses, perceptions of culture sensitivity prior to departure, hierarchies, abuse and objectification in the clinic, communicative challenges in the clinic, increased awareness of different nursing discourses, culture sensitivity experienced in practice, socializing into a new nursing discourse, patients' rights and professional practice and work environment.

### 3.1 Apprehension about conflicting nursing discourses

The first focus‐group interview was conducted prior to departure to Nicaragua. Students discussed the importance of respecting the cultural context and the people in it at any time. They expressed concerns about discussing ethical dilemmas with Nicaraguan nurses. As students, they emphasized that they would have to be cautious how they give their feedback to their supervisors during the clinical placement. They reflected on how receptive their colleagues in Nicaragua would be to their views and understanding of problematic clinical situations. They also discussed other challenges, such as making themselves understood in a context where Spanish is the main language. They underlined that it is important to be aware of how to communicate and be conscious in how to convey the message to avoid offenses. Other aspects they discussed were patients' autonomy and they expressed how lucky they are to work in Norway where resources are abundant.

Some students feared that performing nursing as in Norway, where considerable time and focus are spent on interpersonal relationship with the patients, could lead to conflict with local nursing discourses. They mentioned that, during the nursing education in Norway, there is a strong focus on communication. In addition, they emphasized the importance of being aware of the nonverbal body language because they had learnt that this is extremely important.

### 3.2 Perceptions of cultural sensitivity prior to departure

Before departure to Nicaragua, we explored the students' preconceptions about culture sensitivity. During the first focus‐group interview, we asked them explicitly to share their thoughts on this topic. They all emphasized the importance of being humble and genuinely interested in the persons they meet and be “mentally present” for them. They mentioned the importance not to prejudge other point of views, other ways of performing nursing or other opinions. Some commented that to be keen to know more about Nicaraguan culture is a positive sign of interest. They said that it is important to meet every person as a unique individual, to take into consideration the background of others, because there are also differences in the same culture. One student underscored that it is important: “to be equipped [prepared] when meeting people with a different starting point and give them equal care, even if they have a different view or mind‐set than we have.”

### 3.3 Hierarchies, abuse and objectification in the clinic

After a few weeks in the field, the students wrote their first assignment based on challenges they experienced in the clinic. They faced many ethical challenges and described experiences of a harsher reality than the one they were used to. One student wrote about the breach of confidentiality, one of the ethical guidelines that nurses are committed to:

The nurse tells me that the person who is sitting next to us is an alcoholic and that he has been in and out of an institution. Clearly, he becomes bothered and looks away. I think this is an unpleasant situation and feel very sorry for the poor man that has been designated that way in front of me unnecessarily, because we do not have anything to do with him. I think the power relationship the nurse showed in this case was very abusive and the patient clearly was not able to speak for himself… I feel I have problems with this type of superior talk over the heads of patients…

Some of the students explained this lack of confidentiality by healthcare personnel as cultural:

In Nicaragua, they have a collectivistic vision where the families live together and take care of each other, neighbours and the community. The neighbour knows a lot about you. However, with this, a lot of talk comes along with it. Everyone talks about each other and nothing is left out. I feel that this might influence the culture among nurses and patients.

Some students reflected on how difficult it was to accept that the nurses tend to talk about the patients and share information indiscriminately. These students stated that it is not acceptable to share information about patients without a purpose. Another challenge the students shared with us was relationships with the patients and the apparent lack of focus on the patients' mental health. They commented on how the focus of local nurses was on vital parameters instead of the relationship with the patient: “The focus was mostly on how the mothers should clean, hold and feed their babies.” Several students commented and reflected on young pregnant women's apathetic appearance during their stay in a maternal care facility unit. One wrote: “They seem apathetic, with a blank gaze, show no motions, no joy. Nobody offers support to them, not even their own parents. Who are they supposed to get support from?”

Other issues the students reflected on in their first written assignment were power relations, abuse and awareness of hierarchy systems in the clinical placement. As foreign nursing students, they felt that they had a lower status than their fellow students from Nicaragua, even if they were in the same year of their nursing education. In addition, they pointed out that nobody contradicts health personnel with higher status and qualification than your own.

During their clinical placement, all the students experienced similar ethical challenges and observed situations they felt were bothersome and challenged their values and norms as future nurses. One student expressed the difficult balancing between being a humble silent observer and expressing open dissent:

… during my clinical placement, I have experienced situations that for me were very difficult… Although I feel one has to be humble and careful as a guest in this country, there are situations where one is required to tell that one doesn't agree…

Some of the students wrote that when their ethical values are challenged, it is more difficult for them to try to be empathetic towards the Nicaraguan nurses and share their vision and understand their culture. One student pointed out that even if she had tried to prepare herself before going to Nicaragua, the reality that struck her during clinical placement was very different when she found herself faced with ethically challenging situations. The ethical challenges were many and students shared many stories and observations through their written assignments:

I could probably write a thesis about ethical issues. In many situations, confidentiality seems completely absent. What I reacted most to, in relation to ethics, is probably the way the patient tend to be objectified in many situations … In one of the many situations I observed, a caesarean [section] take place where the mother was a 14‐year‐old girl. It took almost two hours after surgery before the mother could see her baby. She was not able to breastfeed her baby in the beginning … When we asked if it isn't important that the mother stays with her newborn baby right away, they responded that it is very important. But in this particular case the mother was only 14 years old and it was not the nurses' fault that she had become pregnant without knowing how to breastfeed. It was not her job to teach this girl to breastfeed, so she had herself to blame. All this was said when the girl's mother listened. It is terrible, but I really wanted to shake that nurse [physically]. What did she know about how this little girl had become pregnant? When we talked to our contact person [Norwegian students have a local nurse working at NN, a facilitator who they meet once a week, to help them reflect on different challenges they have experienced during their clinical placement] about this situation later on, she confirmed that, unfortunately, such attitudes are common among nurses. Many local nurses feel that young mothers can only blame themselves and that they are a huge burden for the healthcare system.

Another student wrote about how health personnel can get angry with the woman giving birth if they do not push hard enough. Local nurses tell these girls that the pregnancy is their own fault and often ignore the patient when they express pain. On the contrary, the students claimed that when they acknowledge patients as fellow human beings and treat them empathetically, they experienced a lot of positive feedback from them, even after small measures of acknowledgement. One student wrote:

Here I have discovered that my way of meeting people is good for them; a friendly appearance, a smile and a handshake. Often this is all it takes, to create a relationship and trust. I have met people that have enjoyed sharing their experiences and feelings with me and they wanted to listen to me telling [about myself]. Most of them are willing to listen and tell [about themselves], even if I share only a little of myself. They [usually] get little attention … and it has been a pleasure to experience their joy after giving so little. This little measure is just what I would like to promote in a workshop [with local nurses].

### 3.4 Communicative challenges in the clinic

After almost 10 weeks of clinical placement, the students wrote their last assignment with focus on their learning outcomes and reflections about their own expectations during clinical placement. All of the students reflected on challenges concerning communication, how to understand their colleagues, the patients and next of kin. Even though they experienced many challenges concerning communication in another language, all of them concluded that they had a valuable experience in how it feels to be in a foreign country.

Experiencing and struggling with language barriers was clearly an issue they all had in common. However, they also expressed that they learnt how to cope:

Despite difficulties with communication, I think got a lot out of my experience during clinical placement. I had to adjust to the way they perform nursing, due to their culture, be humble and interested in learning their methods, but also stand up for the things we stand for and present them when convenient, without stepping on someone's toes.

Some students also wrote about feeling invisible and how they felt some nurses avoided them because their lack of communication skills in Spanish. One student wrote:

The feeling of not being seen, not being able to contribute and on many occasions we only “tail behind” the nurses here is probably the hardest thing to swallow. One wants to do things, show that one can [accomplish], get a sense of achievement—yes; simply contribute!

Many of the students expressed that their limited language skills created challenges in the clinic. There are limits for how many times a nurse can bear to repeat herself to make students understand.

### 3.5 Increased awareness and different nursing discourses

In their written assignments, the students related their experiences of another nursing context and a different nursing role. Many counter‐intuitive situations they observed made them reflect on their own nursing role and other ways of providing health care. One student wrote:

I have learned about the nursing role in Nicaragua, for better or worse. Burnout can be one of the explanations why the nurses are not very friendly to the patients. … If you must live with a constant anxiety every day [the students understood, learned and observed that many nurses had long working hours, many patients to take care of, low salaries and sometimes problems at home because of their working hours], it is clear that you don't have the energy to take care of others. Nevertheless, this does not justify their behaviour, but it gives me a better understanding of why things are the way they are. To appear cold and with little empathy can never be justified … the patients were often passive recipients, many of them cannot write or read, so they do not have another choice than to do what the doctors and nurses tell them to do.

Another student wrote: “I was prepared that it could be challenging to meet the different fates here, but I was not prepared facing the harsh mentality among some of the health personnel here.” The student later explained how hard it is for health personnel in Nicaragua because of the long working hours. Clearly that made an impression on the student. Another student also commented on this topic:

Often women here have many children and they struggle to make ends meet. All these factors can lead to a lack of interest and a feeling of indifference towards the patients… the nurses told us that if they spend a lot of time and effort on helping the patients out of bed, they would ruin their backs, all of them. In a way, this is true, but it could not occur to me as a nurse in Norway, to leaving a patient in bed after a wound care.

Some students emphasized the high level of skills concerning different procedures among Nicaraguan nurses. However, they all said they got the impression that there is a lack of interest, compassion and sympathy for patients among Nicaraguan nurses. Students also found the Nicaraguan nursing context very different from their own nursing context in the Norwegian healthcare system. In Nicaragua, the next of kin deliver most of the physical and emotional care for the patient and the nurse mainly focus on procedures, instrumental care and administration of medication. One student wrote about how heavy‐handed the local nurses can be towards patients. She never observed nurses sitting beside a patient asking the patient how he/she was doing and feeling. One student wrote:

Nurses in this country for example cannot rely on a health personnel law that functions. With over 40 patients divided between two nurses, it is understandable that they cannot conduct emotional care, mobility care in bed and feeding all patients. Neither do they have a system that supports them if their dire working conditions cause back injuries and such. In addition, this is something I have not taken into account earlier. There is also a huge amount of statistics, as they also get funding from the World Health Organization. This paperwork is extensive and on many occasions, we asked why they do this paperwork instead of spending time with the patients.

At the same time, the local nurses impressed them with their skills in certain procedures and they understood how difficult their life could be as a nurses and employees. Nevertheless, they all had difficulties executing nursing in an environment where they perceived that the health care lacked the compassionate dimension.

### 3.6 Culture sensitivity experienced in practice

In the second written individual assignment, students were asked to reflect on their experiences from the clinical placement and their achievement of learning objectives. One of the central learning objectives of the students was culture sensitivity. One student wrote: “It [culture sensitivity] has challenged me to try to understand why they [Nicaraguan nurses] practice the way they do and why they react the way they do. This has probably increased my culture sensitivity.” Another student wrote: “I show culture sensitivity in the form of respect for the way they practice nursing here, even though there are things I cannot agree with. Also there are patients from different ethnic groups, who come to receive health care.” Furthermore, another quote from different student:

For me, cultural competence is something you develop through experience, knowledge and developing other tools such as communication … Language and communication are essential to become cultural competent… Cultural competence requires a mixture of openness to new ideas and different ways of doing things and at the same time gaining knowledge through active learning and experience …

Some stressed that the importance of respect and recognition, even if one does not master the language as well and does not know much about the culture and habits of the person one meets. Some of the students emphasized that it is important not to accept all the situations one experiences, but it is important to rationalize one's own feelings to get an understanding: “Without an understanding we cannot see possibilities to start a dialogue, which can be the starting point for change.”

### 3.7 Socializing into a new nursing context

Few days after returning home to the Norwegian context, we completed the second focus‐group interview. Students continued to elaborate and discuss their experiences related in their individual written assignments. They discussed how they gradually felt dragged into a nursing practice dominated by the norms and rules of the Nicaraguan cultural context. They found themselves resisting less and adapting to procedures and practices of the local nurses, even though this conflicted with what they perceived as proper nursing. Some of the students were afraid to offend the local nurses in the units and healthcare centres where they had their clinical placement. This fear adversely affected their attitudes towards the patients. It seems as if they experienced a conflict between how to relate to the local nurses and how to relate to the patients. Students had trouble being faithful to the caring nursing perspective that they have learnt in their Norwegian bachelor training.

### 3.8 Patients' rights and professional practice

Students reacted to their observations of patients' suffering, the harsh attitudes expressed by the local nurses and their actions. The lack of care that the students perceived in the local nursing practice clearly made a huge impression on them. They observed young mothers who did not receive necessary guidance to breastfeed their newborn children, lack of information concerning patient rights and lack of care for young mothers.

Many of the students reflected on what they could have done during their clinical placements. One of the concerns was giving more information to the patients about their rights, because they perceived that local health personnel did not inform them properly about these rights (Normas jurídicas de Nicaragua 2002). They discussed how important it was to inform the patient about their rights and how this might increase the demand for higher healthcare standard in the hospital. They all agreed about the value of their experience of feeling like strangers in another country and their experience of being foreigners and being discussed by local nurses. They emphasized the importance of being conscious about their own professional practice and standards and how they wanted to practice as professional nurses and fellow human being in the future.

### 3.9 Work environment

Some students discussed that how the unsatisfactory nursing they had experienced in Nicaragua was due to economic challenges and burnout among the local nurses. However, some students disagreed. These students pointed out that a friendly smile and care does not cost money or take much of one's time. Nevertheless, all the students agreed that the challenges of the local nurses were long working hours, huge responsibilities, many patients, low payments, unpaid sick leave, problems in supporting their families and lack of recognition for all the work they did. One student said:

I do not necessarily believe that they are bad [persons] or something like that. However, instead of stopping and saying “hello,” “how are you?” or something like that … they just walk straight passed you. Thus, sometimes I think it is not only what they did that was so terrible …, but also what they did not do …

The students expressed how impressed they were because the nurses always were creative in solving challenges caused by a lack of equipment and resources.

Students experienced another nursing role and another way of giving health care in Nicaragua. They observed that some patients got care from their next of kin and not from the nurses. They experienced that several nurses used much of their time on documenting and less on the patients. However, they also experienced the relationship between nurses and clinical doctors to be warm and full of care. Another student said:

It seems like they [the local nurses] have some kind of power relationship were they are supposed to look down on the patient, or that they have a very cold façade where they are the ones who direct, determine and know best.

Some of the students also mention how the nurses work without much resources and how they have learnt to be more solution oriented working in an environment with limited access to equipment and resources in the hospital, for instance, gloves, soap, medicine, disinfectant gel, clean water and other items.

## 4 INTERPRETATION AND DISCUSSION

Springer and Clinton invoke a Foucauldian discourse analysis and emphasize the importance of nurses, nursing scholars and nursing students asking critical questions about nursing knowledge and its organization (Springer & Clinton 2015, p. 96). We apply the concept discourse coined by Foucault to explore power relations and the contested nursing discourses encountered by Norwegian bachelor nursing students.

Above, we have uncovered and illustrated some manifestations of power and resistance through various contested nursing discourses encountered and experienced by bachelor nursing students during their clinical placement in Nicaragua. Data collected at various stages during students′ learning trajectories illustrate how they gradually gained awareness of the Nicaraguan nursing discourses they encountered. In addition, students gradually became aware of Norwegian nursing discourses they, initially unknowingly, brought with them into their clinical placement. The students′ growing awareness of these nursing discourses already appears in the first focus‐group interview conducted prior to their departure to Nicaragua. At this point, students reproduced Norwegian nursing discourse as a tacit ideology for proper nursing care. This implicit ideology became apparent and surfaced as students expressed their expectation and apprehensions about possible challenges they would encounter with Nicaraguan nursing discourses during their clinical placement in Nicaragua. Already at this stage, students anticipated possible clashes between Norwegian nursing discourses and Nicaraguan nursing discourses. Students pointed out that they should be careful about how they presented their feedback and critical views about Nicaraguan nursing discourses during clinical placement.

In our study, “culture sensitivity” is both an empirical fact, an ideology, and an analytical approach. Culture sensitivity is an empirical fact, as it is one of the learning objectives of the Norwegian bachelor nursing students. Culture sensitivity is also an analytical approach, which can enhance reflection among students. Furthermore, from our introduction and our data above, it is evident that culture sensitivity is a well‐established social and political stance in Norway, an intrinsic part of the Norwegian welfare state ideology. More specifically, it is also evident that culture sensitivity is a guiding principle of contemporary humanistic Norwegian nursing discourse. The ideal interpretation and application of culture sensitivity, which students had learnt as part of their nursing curriculum, was often difficult to put into practice during their clinical placement abroad as it could lead to clashes with the Nicaraguan nursing discourses. At the same time, their resistance and the clashes between nursing discourses made students increasingly aware and sensitive about the Norwegian nursing discourses they had learnt and been socialized into. Students underscored that it is easier to practice culture sensitivity in Norway, where this ideology is advocated and where resources are abundant. Nevertheless, students emphasized that resistance, clashes and conflicts may occur in Norway as nurses strive to practice in accordance with a culture sensitive ideology.

Students searched for various explanations of the Nicaraguan nursing behaviour they experienced among Nicaraguan nurses. In their written assignments and in the second focus‐group interview, students pointed out that many of the Nicaraguan nurses seemed to have a huge workload. They thought that this affected Nicaraguan nurses' attitude and care towards the patients. In some hospitals, a Nicaraguan nurse may have up to 12 hour shifts and the responsibility for many patients. Other challenges for nurses in Nicaragua are inadequate social security and health insurance. The students mentioned that the local nurses had informed them that the poor health insurance made them avoid helping patient out of bed in some cases. Some students agreed that some Nicaraguan nurses possibly suffered from burnout syndrome.

The students reacted strongly on what they perceived as violence against patients. For a wider understanding of this phenomena, we use the definition violence provided by d`Oliviera, Diniz, and Schraiber (2002), focusing on several types of violence such as neglect, verbal violence, including rough treatment, threats and scolding, shouting and intentional humiliation, physical violence, including denial of pain relief when technically indicated. At the Caribbean coast of Nicaragua, many people live in economic poverty, as it is the poorest regions in the country, RAAS and RAAN. We cannot conclude that nurses at the Caribbean coast suffer from burnout. However, there are ample indications of mental fatigue, cynicism and reduced personal efficacy among some local nurses reported by students (Laschinger & Fida, 2013). Burnout is a combination of negative attitudes and chronic exhaustion. Chronic burnout is considered an important moderator of daily employee functioning (Bakker & Costa, 2014).

In a recent study based on interviews with 35 black, low‐income South African women who gave birth in a public health facility, Rachelle Chadwick (2017) reports about their experiences of obstetric violence. Her analysis of abuse of women and girls during childbirth is framed within a Foucauldian approach to power and using the concept of assemblage. Chadwick argues that obstetric violence needs to be conceptualized as a mode of discipline embedded in the normative relations of class, gender, race and medical power. In the South African public health sector, these normalized “birth assemblages” are shaped by racialized, classed, gendered and medicalized norms which often function as moral imperatives (Chadwick, 2017, pp. 16 – 17). Chadwick′s multifaceted reading of obstetric violence helps illuminate the experiences related by the Norwegian nursing students from the Nicaraguan setting.

The current Norwegian nursing educational paradigm has a strong focus on academic, analytical, assertive and reflective qualities. The educational framework for the Norwegian students' clinical placement in Nicaragua is geared towards fostering the analytical, assertive and reflective qualities. Above we have described the framework, process and “technologies,” which facilitate the students' reflection and professional growth. Gilbert (2001) and Fejes (2008) have used a broad Foucaldian perspective and criticized the current widely advocated so‐called reflective practice in clinical supervision. These authors describe reflection as “confession” and an instance of political ambition by nursing supervisors to govern and shape certain nursing discourses and “subjectivities” based on current nursing ideologies in vogue. These authors claim that reflection is not a neutral apolitical practice, but a powerful governing practice, which reproduces desirable discourses. Gilbert and Fejes claim that these supervision technologies are modes of surveillance that discipline nurse professionals and students into autonomous and self‐regulating subjects (Fejes, 2008; Gilbert, 2001). In other words, supervision technologies such as focus‐group discussions and reflective written assignments are tools with which bachelor nursing supervisors police and reproduce the Norwegian nursing discourse among students.

Rolfe (2006) has responded to Gilbert's critique with an alternative analysis of reflective practice in clinical placement supervision. Rolfe claims that reflective practice as an aspect of clinical supervision can be seen as an actively emancipatory epistemological project. Here, the focus is not on students' personal confession, a reproduction of nursing ideology, but on the improvement of student nursing practice based on reflection about their clinical experiences. Rolfe underscores that the knowledge generated by the individual practitioners' reflection on their own practice promotes them to the status of enlightened “researchers and theorists” in their own right (Rolfe, 2006, pp. 599–600).

### 4.1 Limitations and recommendations for further study

This study has several limitations. Data material was collected from a limited number of students. The study did not include interviews with Nicaraguan nurses and detailed data about the broader context of the nurses working conditions. Nevertheless, the study uncovered a need to explore the origin of Nicaraguan nursing discourse further to gain a greater understanding of local nurses' attitudes and actions. Our data showed that the Norwegian students experienced how Nicaraguan nurses were overworked and stressed due to a heavy workload, extensive responsibilities and long working hours. There is a need to explore the working conditions of the Nicaraguan nurses further in a future study. Such a study can uncover issues and knowledge of their working conditions, which may affect their ability to deliver patient care.

### 4.2 Concluding remarks

Students expressed increasing awareness and reflections about the power relations shaping clinical encounters and nursing discourses throughout their learning trajectory in clinical placement. They became more aware about the politics of various nursing discourses through their experiences of conflicts and their resistance. Their awareness and reflexion were born during their oscillation between being judgemental “ethnocentric” and accepting “cultural relativistic”. Their realization process took considerable time. Students told us that there was a limit to how much they could be prepared prior to their travel to Nicaragua. However, to some extent, they could be equipped before they left through preparatory courses and workshops. As the students' awareness matured, they felt better prepared to face clinical challenges in contemporary multicultural Norway. Students agreed that with their experiences from clinical placement in Nicaragua, they were better armed to tackle various challenges they expect to meet in the clinic in their future work as professional nurses. During the regular response and audit, the students recommended that the focus‐group interviews should be offered as standard option for all students who do clinical placement abroad. Students experienced the focus‐group sessions as a valuable opportunity to pre‐brief, debrief and reflect.

## ACKNOWLEDGEMENTs

We thank the students for their cooperation in this study.

## CONFLICTS OF INTEREST

None declared.
